# Meta-analysis and meta-regression of omega-3 polyunsaturated fatty acid supplementation for major depressive disorder

**DOI:** 10.1038/tp.2016.29

**Published:** 2016-03-15

**Authors:** R J T Mocking, I Harmsen, J Assies, M W J Koeter, H G Ruhé, A H Schene

**Affiliations:** 1 Program for Mood Disorders, Department of Psychiatry, Academic Medical Center, University of Amsterdam, The Netherlands; 2 Department of Psychiatry, Mood and Anxiety Disorders, University Medical Center Groningen, University of Groningen, Groningen, The Netherlands; 3 Department of Psychiatry, Radboud University Medical Center, Nijmegen, The Netherlands; 4 Donders Institute for Brain, Cognition and Behavior, Radboud University, Nijmegen, The Netherlands

## Abstract

Omega-3 polyunsaturated fatty acid (PUFA) supplementation has been proposed as (adjuvant) treatment for major depressive disorder (MDD). In the present meta-analysis, we pooled randomized placebo-controlled trials assessing the effects of omega-3 PUFA supplementation on depressive symptoms in MDD. Moreover, we performed meta-regression to test whether supplementation effects depended on eicosapentaenoic acid (EPA) or docosahexaenoic acid dose, their ratio, study duration, participants' age, percentage antidepressant users, baseline MDD symptom severity, publication year and study quality. To limit heterogeneity, we only included studies in adult patients with MDD assessed using standardized clinical interviews, and excluded studies that specifically studied perinatal/perimenopausal or comorbid MDD. Our PubMED/EMBASE search resulted in 1955 articles, from which we included 13 studies providing 1233 participants. After taking potential publication bias into account, meta-analysis showed an overall beneficial effect of omega-3 PUFAs on depressive symptoms in MDD (standardized mean difference=0.398 (0.114–0.682), *P*=0.006, random-effects model). As an explanation for significant heterogeneity (*I*^2^=73.36, *P*<0.001), meta-regression showed that higher EPA dose (*β*=0.00037 (0.00009–0.00065), *P*=0.009), higher percentage antidepressant users (*β*=0.0058 (0.00017–0.01144), *P*=0.044) and earlier publication year (*β*=−0.0735 (−0.143 to 0.004), *P*=0.04) were significantly associated with better outcome for PUFA supplementation. Additional sensitivity analyses were performed. In conclusion, present meta-analysis suggested a beneficial overall effect of omega-3 PUFA supplementation in MDD patients, especially for higher doses of EPA and in participants taking antidepressants. Future precision medicine trials should establish whether possible interactions between EPA and antidepressants could provide targets to improve antidepressant response and its prediction. Furthermore, potential long-term biochemical side effects of high-dosed add-on EPA supplementation should be carefully monitored.

## Introduction

Omega-3 polyunsaturated fatty acids (PUFAs) have been proposed as a treatment for major depressive disorder (MDD). Over the last decade, several meta-analyses have been performed, which suggested variable degrees of beneficial effects of omega-3 PUFAs for MDD, but made critical remarks regarding the quality of the evidence and possible publication bias.^1, 2, 3, 4, 5, 6^ These meta-analyses evoked academic correspondence, discussing the used inclusion criteria and selection of outcome measures.^7, 8^ In brief, this correspondence suggested beneficial effects (1) if a higher ratio of omega-3 PUFA eicosapentaenoic acid (EPA) to docosahexaenoic acid (DHA) was being supplemented^7^ and (2) only in patients with actual MDD as opposed to subjects with merely depressive symptoms.^8^

Of these meta-analyses, only the three most recent meta-analyses covered the research performed over the last 5 years: (1) Grosso *et al.*^9^ included randomized controlled trials (RCTs) published up to August 2013, and concluded that omega-3 PUFAs were effective in depressive patients with or without an MDD diagnosis; (2) Yang *et al.*^10^ pooled RCTs published up to March 2015 on the effect of the combination of EPA and DHA on depression in women and observed a beneficial effect; and (3) Appleton *et al.*^4^ performed a Cochrane meta-analysis of RCTs published up to May 2015, concluding that there is a small-to-modest, non-clinically beneficial effect of omega-3 PUFAs.

Importantly, these meta-analyses seem to have several issues of interest. First, they did not correct for potential publication bias, for example, by using the trim-and-fill method.^11^ Second, Grosso *et al.* included two comparisons of the study of Jazayeri *et al.*^12^ This study had three arms using a double-dummy technique (EPA+placebo vs EPA+fluoxetine vs fluoxetine+placebo). Although other meta-analyses only included the fluoxetine+EPA vs fluoxetine+placebo comparison (that is, EPA vs placebo in an add-on design), Grosso *et al.* additionally included the EPA+placebo vs fluoxetine+placebo comparison (that is, EPA vs fluoxetine during additional placebo treatment). Thereby, Grosso *et al.* not only compared EPA vs placebo, but additionally included a comparison of EPA vs active antidepressant medication, potentially reducing the overall effect size. Third, Grosso *et al.* and Yang *et al.* included three articles that all seem to report on the same RCT,^13, 14, 15^ that is, (partial) duplicate publication which distorts pooled-effect estimates. Fourth, Grosso *et al.* and Appleton *et al.* missed potentially important evidence by not including all RCTs on MDD patients in their analysis (for example, Lesperance *et al.*^16^ in Grosso *et al.*; and Mozaffari-Khosravi *et al.*^17^ in Appleton *et al.*). Although the study of Mozaffari-Khosravi *et al.* included mild-to-moderate MDD patients, all patients fulfilled MDD criteria, baseline severity was comparable to other included studies and other included studies (for example, Lucas *et al.*^18^) also used an upper limit for MDD severity. Fifth, all three meta-analyses only used post-intervention effect size data, while repeated measures are available, which may improve effect size estimation precision. Sixth, by including RCTs that not only included MDD patients but also patients with dysthymia,^13, 14^ all three meta-analyses did not specifically focus on MDD only. In addition, Appleton *et al.* included the trial by Peet and Horrobin^19^ that did not use MDD diagnosis as an inclusion criterion, but rather a cutoff score on a depression symptom questionnaire. Moreover, Appleton *et al.* and Grosso *et al.* also included trials on subjects with MDD secondary to neurological or other somatic disorders (for example, Da Silva *et al.*),^20^ which may increase heterogeneity due to different underlying pathophysiology.

Therefore, our study aims at continuing the academic debate by performing a revised updated meta-analysis on the effectiveness of omega-3 PUFAs for the treatment of MDD taking into account the above issues of concern. In addition, we performed meta-regression analyses to explain expected heterogeneity, by testing the effects of EPA and DHA dose and their ratio, study duration, participants' age, percentage antidepressant users, baseline MDD symptom severity, publication year and study quality.

## Materials and methods

### Search strategy

We performed a search in the Medline and Embase databases (from inception up to 8 December 2015). We used a search strategy combining terms regarding MDD and fatty acid supplementation using Boolean operators (See Supplementary Methods). We additionally searched references of selected studies and earlier meta-analyses for potential studies.^1, 4, 9^

### Study selection

Two independent reviewers (RJTM and IH/JA) screened the identified articles for their relevance by title and abstract, and—if necessary—the full text. In case of disagreement between reviewers, inclusion could be conclusively determined by discussion in all the cases. *A priori* criteria for inclusion of studies were: (1) design was a randomized placebo-controlled trial; (2) inclusion of adult patients with MDD according to the Diagnostic and Statistical Manual of Mental Disorders (DSM) as assessed by a standardized clinical interview. We excluded studies that (1) specifically studied perinatal or perimenopausal MDD or (2) MDD secondary to other neuropsychiatric disorders.

With these in- and exclusion criteria, we aimed to test the effect of omega-3 fatty acid supplementation on depressive symptoms specifically in MDD, as opposed to depressive symptoms in the general population (including subclinical depression). Because previous meta-analyses included clinically more heterogeneous populations which theoretically may preclude meta-analysis, we aimed to prevent inclusion of trials where supplementation of omega-3 fatty acids could improve an underlying somatic or (neuro)psychiatric disorder, with a secondary effect on the depression scores. In addition, by excluding specific subgroups like perinatal or perimenopausal MDD, with a potentially different pathophysiology, we aimed to further minimize heterogeneity among included studies. Because of the widespread comorbidity between MDD and cardiovascular disease, we did not exclude studies that specifically studied MDD with comorbid cardiovascular disease or diabetes.^21^ Nevertheless, we performed sensitivity analyses without the studies that specifically studied MDD with comorbid cardiovascular disease or diabetes.^22, 23^

### Critical appraisal

We assessed the quality of the studies that were selected with the in- and exclusion criteria above using the Jadad scale for reporting RCTs.^24^

### Data extraction

Two independent reviewers (RJTM and IH) extracted the data into specifically designed spreadsheets. We collected the data on descriptives, methods and outcomes. Possible disagreements were resolved by discussion with a third independent reviewer where necessary (JA).

We used the primary outcome measure reported by the study. We used the unadjusted intention-to-treat repeated-measures statistics where available. If multiple primary outcomes were reported, we used the Hamilton Depression Rating Scale (HDRS).^25^ If unavailable, we used another clinician-rated outcome. If unavailable, we used a self-rated depression scale. We performed sensitivity analyses using unpublished additional data derived from the authors where available.^4, 12, 26, 27^

### Statistical analysis

We performed the statistical analyses with Comprehensive Meta-Analysis version 2. We used standardized mean differences as the summary statistic for meta-analysis. We assessed heterogeneity by calculating the *I*^2^ statistic.^11^ Given the expected heterogeneity, we *a priori* used a random-effects model.^11^ We performed the sensitivity analyses using fixed-effects models because they are considered to be less vulnerable for publication bias. We assessed publication bias by plotting a funnel plot, and reporting the classic and Orwin's fail-safe N, Begg and Mazumdar rank correlation and Egger's regression intercept.^11^ Finally, we adjusted the values using the Duval and Tweedie's trim-and-fill method.

We performed meta-regression using the method of moments computational option.^11^ We *a priori* planned to test the univariate effects of EPA and DHA dose, study duration, participants' age, percentage antidepressant users, baseline MDD severity, publication year and study quality, on the effect size of trials. To compare EPA- and DHA-dose effects for studies where multiple subgroups were available, we regarded these subgroups as independent, assuming independence where necessary.^11^ For baseline severity, we converted scores to the 17-item Hamilton Depression Rating Scales using available conversion tables where needed.^28, 29^

## Results

### Selection of studies

The search strategy resulted in 1955 articles for consideration in the present meta-analysis. The studies were excluded for several reasons, including not applying a randomized placebo-controlled trial design, no inclusion of subjects with MDD as ascertained by a structured clinical interview,^30, 31, 32, 33^ or specifically including patients with perinatal or perimenopausal MDD, or MDD secondary to other neuropsychiatric disorders^20, 34^ (Supplementary Figure S1). After applying the in- and exclusion criteria, we included 15 studies,^12, 16, 17, 18, 22, 23, 26, 35, 36, 37, 38, 39, 40, 41, 42^ including a total of 1253 MDD subjects. Study-characteristics are provided in Supplementary Table 1. Of these 15 studies, 1 small preliminary study reports unpublished data (Coryell *et al.*, related to Fiedorowicz *et al.*^42^), and 1 small study has a considerable drop-out rate (>50%).^41^ Given the relatively preliminary nature of these two studies, we included them only in separate sensitivity analyses.

### Meta-analysis

**Figure 1:**
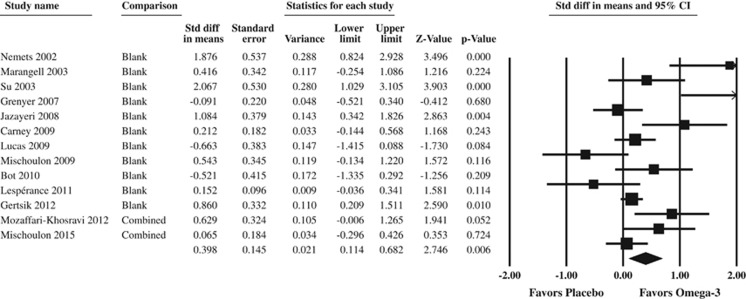
Forest plot of the meta-analysis on the effect of omega-3 fatty acid supplementation on depressive symptoms in major depressive disorder. For the comparison column, ‘blank' means that there was only one comparison in the study, ‘combined' means that different comparisons were combined for an overall study estimate. Studies are sorted by publication year.

The meta-analysis on 13 RCTs in 1233 MDD subjects showed an overall beneficial effect of omega-3 PUFAs on depressive symptoms in MDD (standardized mean difference=0.398 (0.114–0.682), *P*=0.006, random-effects model, Figure 1). For the sensitivity analyses, see Supplementary Results.

### Publication bias

The funnel plot for all the available data is shown in Supplementary Figure 2. The classic fail-safe N was 95, Orwin's fail-safe N was 19 with criterion for a ‘trivial' standardized difference in means as 0.1 and mean standardized difference in means in missing studies as 0. This implied that at least 19 studies without any effect must have been left unpublished to reduce the overall effect to a trivial effect. Regarding the Begg and Mazumbar rank-correlation test, Kendall's taus with and without continuity correction were 0.21 (*P*_2-sided_=0.28), and 0.22 (*P*_2-sided_=0.26), respectively, indicative of no publication bias. Egger's regression intercept was 1.13 (95% confidence interval: −0.63 to 2.91; *P*_2-sided_=0.19), also suggesting no significant publication bias. Duval and Tweedie's trim-and-fill method used the random-effects model looking for missing studies to the left of the mean, that is, less positive effect of supplementation, and showed that no studies needed to be trimmed. See Supplementary Results for sensitivity analyses.

### Meta-regression

Method-of-moments meta-regressions were performed to explain significant heterogeneity (*Q*=45.04, *P*<0.001, *I*^2^=73.36). Higher EPA dose (*β*=0.00037 (0.00009–0.00065), *P*=0.009), higher percentage antidepressant users (*β*=0.0058 (0.00017–0.01144), *P*=0.044) and earlier publication year (*β*=−0.0735 (−0.143 to −0.004), *P*=0.04) were significantly associated with a better outcome for PUFA administration, whereas DHA dose (*β*=0.00015 (−0.00029 to 0.00059), *P*=0.498), EPA/(EPA+DHA) ratio (*β*=0.0044 (−0.0034 to 0.012), *P*=0.272), study duration (*β*=−0.012 (−0.026 to 0.002), *P*=0.086), baseline severity (*β*=−0.00023 (−0.14 to 0.14), *P*=0.997), age (*β*=−0.036 (−0.075 to 0.002), *P*=0.06) and Jadad score (*β*=−0.0791 (−0.327 to 0.169), *P*=0.531) were not significantly associated. See Supplementary Results for the sensitivity analyses.

## Discussion

Our revised updated meta-analysis adds nuance to the continuing debate on the effect of omega-3 PUFA supplementation on depressive symptoms in MDD. By addressing several issues as noted in the introduction, the present meta-analysis studied effects of omega-3 PUFA supplementation in all available evidence in a specific relatively homogeneous group of MDD subjects. Overall, with an standardized mean difference of 0.398 present meta-analysis shows a beneficial effect of omega-3 PUFAs that is comparable to effects reported in meta-analyses of antidepressants.^43^ Of note, this effect seemed larger in studies (1) supplementing higher doses of EPA and (2) performed in patients using antidepressants (augmentation/add-on), while it was independent of baseline depression severity or EPA/(EPA+DHA) ratio. However, more recent trials had smaller effect sizes, independent of trial quality.

By including a relatively homogeneous group of patients with MDD according to DSM criteria as assessed using a standardized clinical interview, we aimed to enhance internal validity and generalizability of the present meta-analysis. Nevertheless, the included MDD patients still form a heterogeneous group including both subjects that will benefit from omega-3 PUFA supplementation and those that experience no or even negative effects.^21^ Several other unreported factors may influence the response to omega-3 PUFA, which could not be tested in the present meta-analysis and meta-regression, for example, measurements of inflammation or nutrigenetics.^44, 45, 46, 47^

Nevertheless, we were able to test sample and trial-related factors that may influence omega-3 PUFA response. In the present meta-analysis, response was independent of baseline MDD symptom severity. This may be because we included a relatively homogeneous sample of MDD patients in comparison with earlier meta-analyses that also included subjects with depressive symptoms.^1^ In contrast to the lack of effect of baseline severity, we noticed that the effects of supplementation seemed larger in patients being treated with antidepressants compared with patients not being concurrently treated with antidepressants. Of note, MDD baseline severity was not associated with percentage antidepressant use across trials (*P*=0.778). Therefore, this larger effect in trials where patients were being treated with antidepressants suggests an interaction between antidepressant use and omega-3 PUFAs at the biological level, for example, due to PUFAs' modulating effect on neuronal membrane–antidepressant interactions or on inflammatory pathways.^21, 45, 48, 49^ In addition, omega-3 PUFAs may interfere with serotonergic neurotransmission,^50^ or antidepressant transport across the blood–brain barrier by influencing p-glycoprotein.^51^ It could be highly clinically relevant to follow up on this finding that omega-3 PUFAs seem to have more effect in studies where participants use antidepressants, by further investigating the interaction between omega-3 PUFAs and antidepressants from (integrated) biological and clinical perspectives.

The present meta-regression finding that higher EPA dose was associated with better response, nuances an earlier meta-analysis that observed significant effects after applying a dichotomous cutoff at 60% EPA content.^7^ First, although it remained relatively unclear how this previous cutoff was derived, our regression model suggested a linear relationship in all the available data without using an artificial cutoff. In addition, the present meta-analysis also showed that EPA/(EPA+DHA) ratio had no significant effect, nor had DHA dose. Altogether, this suggests that it is not the ratio of EPA vs DHA that is important, but rather the higher EPA dose. Although hypothesized, it does not seem that DHA counteracts the effects of EPA (for example, by competition for target proteins or membrane incorporation);^6, 52, 53^ DHA simply has no detectable pooled effect on the MDD symptoms. Nevertheless, it remains surprising that EPA seems to be responsible for the beneficial effects of omega-3 PUFA supplementation while DHA concentrations appear to differ more between patients and controls.^54^ In addition, the regression effect of EPA dose on RCT outcome depended to some extent on one trial that supplemented the highest EPA concentration.^26^ On the basis of these findings, it could be argued that the beneficial effects of omega-3 PUFA supplementation are not because supplementation corrects a membrane DHA ‘deficit', but rather due to the anti-inflammatory characteristics of EPA's oxidation products.^21^ Future mechanistic studies in a more continuous dose range should follow up on these findings.

However, these oxidation products may not have positive effects only. Relatively little is known about the precise role and effect of the great diversity of PUFA oxidation products. Although adverse events reported during the studies were usually mild and gastrointestinal in nature (for example, belching, constipation, fishy aftertaste), exposure to PUFA oxidation products with unknown effects may pose unknown risks in the long term.^21, 47, 55^ It may therefore be advisable to measure these oxidation products during future studies with a longer follow-up to obtain more insight in their potential toxicity. In addition, fishy aftertaste may result in unblinding, potentially distorting effect sizes.^4, 56^

The finding that more recent studies showed smaller effects remains puzzling. It seems independent from study quality, as we observed no association between study effect size and study quality operationalized as Jadad score. It may be that differences in background diet over time contributed to smaller effects of additional omega-3 PUFA supplementation. For example, even though studies usually excluded participants that used omega-3 PUFA supplements on their own initiative at baseline, it may be that participants started using omega-3 PUFA supplements during the study owing to their increasing popularity, thereby diminishing outcome differences in more recent trials. Unfortunately, not enough trials provided uniform data regarding baseline omega-3 PUFA status or intake to formally test this,^57^ which would be interesting in future research.

### Limitations and strengths

Despite several strengths of this meta-analysis, some limitations should be noted. First, the present meta-analysis has not been performed on patient level data. It would be interesting to confirm these findings on a patient level. Moreover, this may also lead to additional predictors of supplementation outcome, giving rise to extra targets for future precision medicine studies. We hope researchers will be motivated to share and pool available data in the future. Furthermore, while the present meta-analysis had enough power to detect small-to-medium effect sizes, smaller regression effects may have been lost as a result of the smaller number of studies due to the specific in- and exclusion criteria. In addition, several concerns regarding the quality of the available evidence could be made,^4^ like in most meta-analyses. Further high-quality evidence to support a beneficial effect of EPA in (antidepressant using) MDD patients could lead to more precise estimates of overall effect size. Finally, to maintain power for the meta-regression regarding the effect of percentage antidepressant users on supplementation outcome, we pooled all concurrent antidepressant classes together, while the effects may differ per class. It would be interesting to further test this in future studies including users of different antidepressants.

Nonetheless, the present study/analyses focused on studies that specifically included patients with MDD according to diagnostic criteria as ascertained with a structured clinical interview. We thus limited clinical heterogeneity while still maintaining a substantial overall sample size. As a second strength, by performing extensive nuanced *a priori* planned publication bias analyses and meta-regression analyses, we revealed new evidence for (1) an effect of EPA dose as opposed to EPA to DHA ratio and (2) an interaction between omega-3 PUFAs and antidepressants, both of which may be highly relevant for clinical practice.

### Conclusion

In conclusion, the present meta-analysis observed a beneficial overall effect of omega-3 PUFA supplementation in patients with MDD according to diagnostic criteria, which seemed larger in studies that supplemented higher doses of EPA and included patients taking antidepressants. Future precision/personalized medicine trials should establish whether possible interactions between EPA and antidepressants could provide targets to improve antidepressant response and its prediction.^48^ Nevertheless, potential long-term biochemical side effects of high-dosed add-on EPA supplementation should be carefully monitored.^21^

## Supplementary Material

Supplementary Methods

Supplementary Results
