# Supplementary material for: Meta-analysis and meta-regression of omega-3 polyunsaturated fatty acid supplementation for major depressive disorder
Source: Transl Psychiatry. 2016 Mar 15;6(3):e756–. doi: 10.1038/tp.2016.29 (PMC4872453; doi:10.1038/tp.2016.29)
Supplement: Supplementary Methods [file tp201629x1.docx]

**Supplemental methods: database searches**

*Search Medline*(depressive disorder [MeSH Terms] OR depression [MeSH Terms] OR depression [Title/Abstract] OR depressive disorder [Title/Abstract] OR depressed mood [Title/Abstract] OR dysthymic disorder [Title/Abstract] OR dysthymia [Title/Abstract]) AND (fish oils [MeSH Terms] OR fatty acids, omega 3 [MeSH Terms] OR Omega-3 [Title/Abstract] OR polyunsaturated FA [Title/Abstract] OR fish oil [Title/Abstract] OR EPA [Title/Abstract] OR DHA [Title/Abstract] OR eicosapentaenoic acid [Title/Abstract] OR docosahexaenoic acid [Title/Abstract] OR alpha-linolenic acid [Title/Abstract] OR cod liver oil [Title/Abstract] OR n-3 fatty acids [Title/Abstract] OR n3 polyunsaturated fatty acids [Title/Abstract])

*Search Embase*

1. exp depression/

2. depressive disorder.mp.

3. depression.mp.

4. depressed mood.mp.

5. dysthymic disorder.mp. or exp dysthymia/

6. dysthymia.mp.

7. 1 or 2 or 3 or 4 or 5 or 6

8. fish oil.mp. or exp fish oil/

9. exp omega 3 fatty acid/

10. omega-3.mp.

11. polyunsaturated fatty acid.mp. or exp polyunsaturated fatty acid/

12. exp icosapentaenoic acid/ or epa.mp.

13. exp docosahexaenoic acid/ or dha.mp.

14. alpha-linolenic acid.mp. or exp linolenic acid/

15. cod liver oil.mp. or exp cod liver oil/

16. n-3 fatty acids.mp.

17. n3 polyunsaturated fatty acids.mp.

18. 8 or 9 or 10 or 11 or 12 or 13 or 14 or 15 or 16 or 17

19. 7 and 18

20. limit 19 to human
