# Supplementary material for: Meta-analysis and meta-regression of omega-3 polyunsaturated fatty acid supplementation for major depressive disorder
Source: Transl Psychiatry. 2016 Mar 15;6(3):e756–. doi: 10.1038/tp.2016.29 (PMC4872453; doi:10.1038/tp.2016.29)
Supplement: Supplementary Results [file tp201629x2.docx]

**Supplemental results: Sensitivity analyses**

*Meta-analysis*

Fixed effects model
Running the overall meta-analysis in a fixed effects model decreased the effect size but increased significance [SMD=0.234 (0.111-0.358), *P*<.001].

Excluding cardiovascular disease and diabetes
Running the analyses without studies that specifically included subjects with comorbid cardiovascular disease^1^ or diabetes^2^ increased both the effect size and significance [SMD=0.502 (0.170-0.834), *P*=.003, random-effects model].

Including preliminary additional studies:
Including one unpublished (Coryell et al., related to Fiedorowicz et al.^3^) and one high drop-out trial^4^ did not substantially change the results [SMD=0.392 (0.120-0.664), *P*=.005, random-effects model].

Including unpublished data:
Including unpublished data from three trials^5-7^ had no remarkable effect on results [SMD=0.353 (0.085-0.621), *P*=.010, random-effects model].

*Publication bias*

Fixed effect model
Running Duval and Tweedie’s trim and fill method using a fixed effect model on all available data to look for studies to the left of the mean resulted in trimming of two studies. The resulting point estimate consequently became smaller, but still significant [SMD=0.172 (0.052-0.292)].

Excluding cardiovascular disease and diabetes
After excluding the studies performed in patients with cardiovascular disease^1^ and diabetes^2^, running Duval and Tweedie’s trim and fill method using the random effects model looking for missing studies to the left of the mean, i.e. less positive effect of supplementation, showed that no studies needed to be trimmed.

*Meta-regression*

Fixed effect model
Overall, fixed effect meta-regression reduced β’s but increased significance, without great differences. Higher EPA-dose [β=0.00026 (0.00008 – 0.00044), *P*=.005], higher percentage antidepressant users [β=0.0041 (0.00011 – 0.00716), *P*=.008] and earlier publication year [β= -0.0469 (-0.085 – -0.008), *P*=.017] remained significantly associated with a better outcome for PUFA-administration, while DHA-dose [β=0.00006 (-0.00018 – 0.00031), *P*=.611], EPA/(EPA+DHA) ratio [β=0.0030 (-0.00096 – 0.00685), *P*=.139], study duration [β= -0.006 (-0.013 – 0.0008), *P*=.085], baseline severity [β= -0.0024 (-0.10 – 0.05), *P*=.542], and Jadad score [β= -0.119 (-0.269 – 0.032), *P*=.122] remained not significantly associated. The effect of age became significant, indicating that in the fixed effects model omega-3 supplementation has less effect in older subjects [β= -0.025 (-0.046 – -0.004), *P*=.017].

Individual study dependency
In order to test to what extent the significant association between EPA-dose and RCT outcome depended on individual study results, we removed each study one by one from the meta-regression. The association always remained significant, except after removal of the study by Su et al., 2003. After removal of this study - that supplemented the highest EPA-dose - the effect estimate remained positive but decreased in size [β= 0.00020 (-0.00019 – 0.00059), *P*=.311].

**References**

1. Carney RM, Freedland KE, Rubin EH, Rich MW, Steinmeyer BC, Harris WS. Omega-3 augmentation of sertraline in treatment of depression in patients with coronary heart disease: A randomized controlled trial. *JAMA - Journal of the American Medical Association* 2009; **302**(15)**:** 1651-1657.

2. Bot M, Pouwer F, Assies J, Jansen EH, Diamant M, Snoek FJ*, et al*. Eicosapentaenoic acid as an add-on to antidepressant medication for co-morbid major depression in patients with diabetes mellitus: a randomized, double-blind placebo-controlled study. *J Affect Disord* 2010; **126**(1-2)**:** 282-286.

3. Fiedorowicz JG, Hale N, Spector AA, Coryell WH. Neuroticism but not omega-3 fatty acid levels correlate with early responsiveness to escitalopram. *Ann Clin Psychiatry* 2010; **22**(3)**:** 157-163.

4. Gonzalez A, Mata S, Sanchez P, Gonzalez D, Urbina M, Fazzino F*, et al*. Omega-3 fatty acids as adjunctive of antidepressant therapy and its effects on brain-derived neurotrophic factor in serum, monocytes and lymphocytes. *Archivos Venezolanos de Farmacologia y Terapeutica* 2011; **30**(4)**:** 72-78.

5. Jazayeri S, Tehrani-Doost M, Keshavarz SA, Hosseini M, Djazayery A, Amini H*, et al*. Comparison of therapeutic effects of omega-3 fatty acid eicosapentaenoic acid and fluoxetine, separately and in combination, in major depressive disorder. *Australian and New Zealand Journal of Psychiatry* 2008; **42**(3)**:** 192-198.

6. Mischoulon D, Papakostas GI, Dording CM, Farabaugh AH, Sonawalla SB, Agoston AM*, et al*. A double-blind, randomized controlled trial of ethyl-eicosapentaenoate for major depressive disorder. *Journal of Clinical Psychiatry* 2009; **70**(12)**:** 1636-1644.

7. Su KP, Huang SY, Chiu CC, Shen WW. Omega-3 fatty acids in major depressive disorder: A preliminary double-blind, placebo-controlled trial. *European Neuropsychopharmacology* 2003; **13**(4)**:** 267-271.
