# Supplementary material for: Meta-analysis and meta-regression of omega-3 polyunsaturated fatty acid supplementation for major depressive disorder
Source: Transl Psychiatry. 2016 Mar 15;6(3):e756–. doi: 10.1038/tp.2016.29 (PMC4872453; doi:10.1038/tp.2016.29)
Supplement: Supplementary Figure 1 [file tp201629x3.docx]

**Supplementary figure S1.** Flow diagram of the in- and exclusion process.

Additional records identified through other sources
(n = 1)

## Identification

Records identified through database searching
Medline (n = 941)

Embase (n=2139)

Full-text articles excluded, with reasons
(n = 18)

- No current MDD according to clinical interview (n = 13)

- Perimenopausal (n = 2)

- Duplicate results (n = 2)

- MDD due to underlying disorder (n = 1)

- No MDD outcome (n = 1)

- No placebo group (n = 1)

*Number may add to more than total if articles fulfil multiple reasons*

Records excluded
(n = 1922)

Studies included in quantitative synthesis (meta-analysis)
(n = 13)

Studies included in qualitative synthesis
(n = 15)

Full-text articles assessed for eligibility
(n = 33)

Records screened
(n = 1955)

Records after duplicates removed
(n = 1955)

## Eligibility

## Included

## Screening
