# Supplementary figures and images for: Meta-analysis and meta-regression of omega-3 polyunsaturated fatty acid supplementation for major depressive disorder
Source: Transl Psychiatry. 2016 Mar 15;6(3):e756–. doi: 10.1038/tp.2016.29 (PMC4872453; doi:10.1038/tp.2016.29)

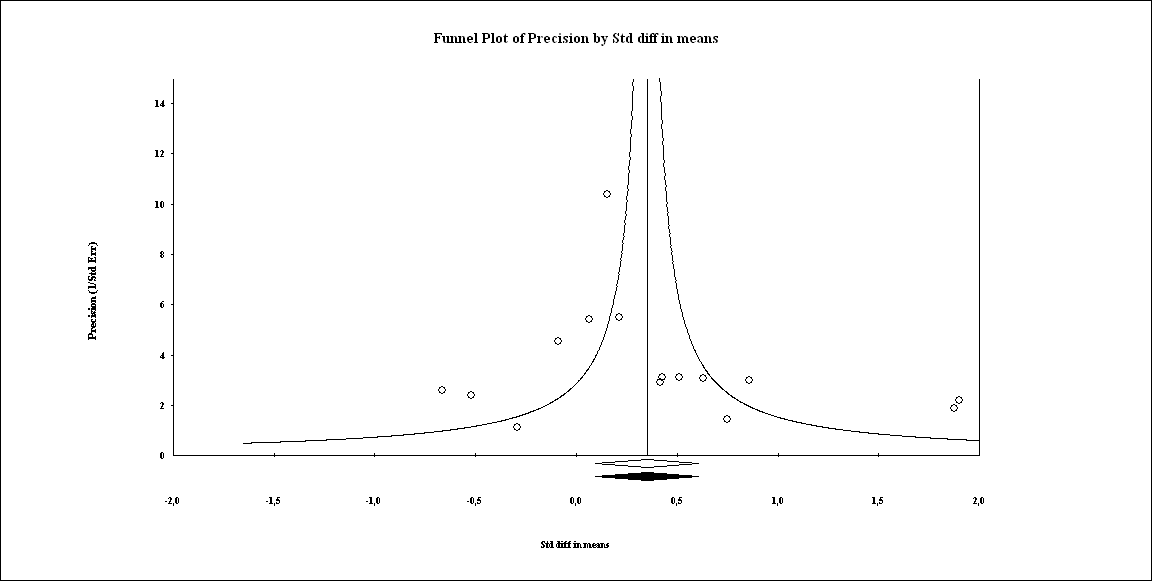

Supplement: Supplementary Figure 2 [file tp201629x4.tif]

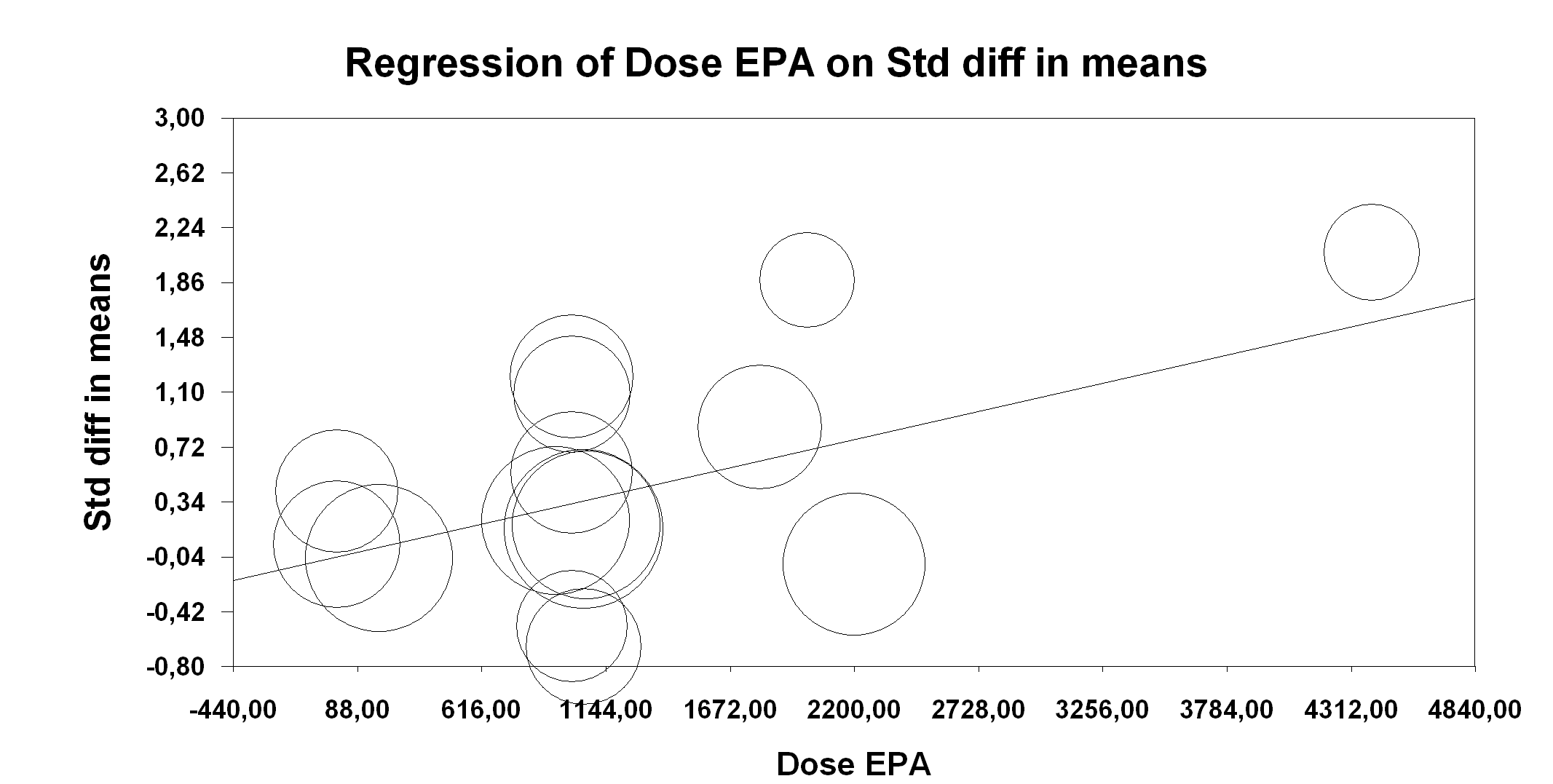

Supplement: Supplementary Figure 3 [file tp201629x5.tif]
